# Postprandial Glycemic and Insulinemic Responses to Common Breakfast Beverages Consumed with a Standard Meal in Adults Who Are Overweight and Obese

**DOI:** 10.3390/nu9010032

**Published:** 2017-01-04

**Authors:** Jia Li, Elsa Janle, Wayne W. Campbell

**Affiliations:** Department of Nutrition Science, Purdue University, 700 West State Street, West Lafayette, IN 47907, USA; li1201@purdue.edu (J.L.); janle@purdue.edu (E.J.)

**Keywords:** breakfast beverage, coffee, dairy, diabetes, glycemic control, insulin, milk

## Abstract

Breakfast beverages with different nutrient compositions may affect postprandial glycemic control differently. We assessed the effects of consuming (1) common breakfast beverages (water, sugar-sweetened coffee, reduced-energy orange juice (OJ), and low-fat milk (LFM)); and (2) fat-free, low-fat, and whole milk with breakfast on postprandial plasma glucose and insulin responses in adults who were overweight/obese. Forty-six subjects (33F/13M, body mass index: 32.5 ± 0.7 kg/m^2^, age: 50 ± 1 years, mean ± SEMs) consumed a standard sandwich with one of the six beverages on separate mornings in randomized order. The test beverages (except water) each contained 12 g digestible carbohydrate. Plasma glucose and insulin concentrations were measured from blood obtained pre- and post-meal at 30-min intervals for 4 h and incremental areas under the curve (AUC) were computed. We found (1) among different beverage types, glucose AUC was higher for coffee versus water, OJ, and LFM. Insulin AUC was higher for coffee and LFM versus OJ and water; (2) Glucose AUCs were not different among water and milks while insulin AUC was higher for milks versus water. In conclusion, consumption of water, reduced-energy OJ, or milk (irrespective of fat content) with a meal may be preferable to consuming sugar-sweetened coffee for glucose control in middle-aged adults who are overweight and obese.

## 1. Introduction

Dietary energy from beverages represents approximately 18% of the total energy consumed by adults in America, according to 2007–2008 National Health and Nutrition Examination Survey (NHANES) data [1]; and energy-containing beverages with different nutrient compositions have differential effects on postprandial glycemia as reflected by the differences in the glycemic index (GI) of the beverages [2,3]. Limited studies exist on the postprandial glucose responses to beverages in the context of a meal. One study compared consuming 500 mL of multiple beverages (water, soy beverage, 2% milk, 1% chocolate milk, orange juice, and a milk-based infant formula) prior to an ad libitum pizza meal on food intake, appetite, and the postprandial glucose response [4]. Concerning the glucose response, the consumption of beverages with the highest sugar content (chocolate milk and orange juice) resulted in the highest plasma glucose concentrations, while 2% milk resulted in the lowest postprandial glucose concentration [4]. These results suggest that beverage consumption prior to a meal may affect postprandial glycemia, but additional studies with alternative designs need to be conducted to corroborate these findings. In particular, a study is needed that utilizes a standard, rather than ad libitum test meal and standardizes the amount of digestible carbohydrate in the beverages.

Therefore, we investigated the effects of consuming iso-volumetric and iso-carbohydrate quantities of four of the commonly consumed breakfast beverages, including water (control), sugar-sweetened coffee, reduced-energy orange juice (OJ), and low-fat milk (1% milk fat) [1] with a standard meal on postprandial glycemia and insulinemia in adults who are overweight/obese and at risk of developing type 2 diabetes (study aim 1). We hypothesized that the meal consumed with low-fat milk would elicit a smaller glycemic response when compared to the meal consumed with coffee or reduced-energy orange juice. Our hypothesis was based on published GIs for milk, table sugar (in coffee), and orange juice (approximately 42, 103, and 81, respectively with white bread as reference) [5] and the observation that the glycemic index of meal components can be used to predict the glycemic response of a mixed meal [2,3]. In addition, we compared three varieties of milk (fat-free, low-fat, and whole milk) to assess the effects of milk fat on the postprandial glycemia and insulinemia (study aim 2). We hypothesized that postprandial glucose and insulin responses would not differ among varieties of milk. This hypothesis was based on the lack of differences in glucose and insulin responses between fat-free milk and whole milk when they were consumed alone [6].

## 2. Subjects and Methods

### 2.1. Subject Recruitment

Subjects were recruited from the greater Lafayette, Indiana, U.S.A. area using advertisements in newspapers, community bulletin boards, and/or community newsletters. Inclusion criteria were: age 35–65 years; BMI 27–40 kg/m^2^; with or without type 2 diabetes; HbA1c < 7.5% (subjects with HbA1c greater than or equal to 7.5% were encouraged to consult their physician); weight stable (±4.5 kg) for the previous 3 months; not currently or within the past 6 months have been following a vigorous exercise regimen or weight loss program; no acute illness; not smoking; not pregnant or lactating or planning pregnancy in the next 3 months; and willing to consume study foods and beverages. Exclusion criteria were: women during the peri-menopause transition (irregular menstrual periods or within 24 months of their last menstrual period); and self-reported lactose intolerance. Fasting-state body mass was measured on each testing day and weight changes of greater than ±3 kg during the study period resulted in dismissal from the study (*n* = 0 were excluded). Subjects were advised to maintain their usual types and levels of physical activity through the study period.

**Figure 1:**
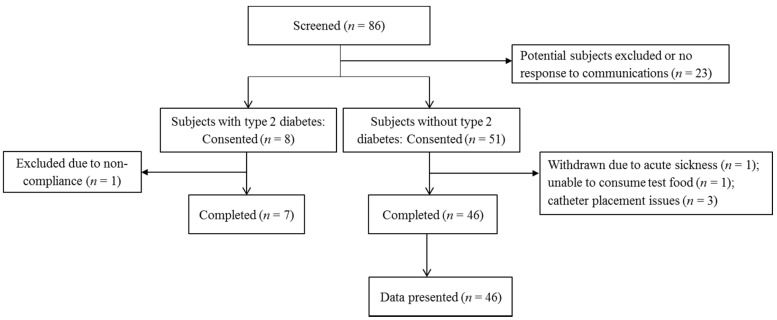
Subject recruitment flow chart.

We initially aimed to recruit 40 individuals (25 with normal glucose tolerance, 15 with prediabetes) without type 2 diabetes and 15 individuals with type 2 diabetes (see sample size estimation section). A priori, our plan was to use the data from the 40 individuals without type 2 diabetes to address our aims, and use the data from the 15 individuals with type 2 diabetes as a pilot investigation. Among the 86 individuals screened, 51 without type 2 diabetes consented and 46 completed the study; the dropout rate was 9.8% (Figure 1).

### 2.2. Subject Screening

At the screening visit, subjects arrived after a 10 to 12-h overnight fast. Body mass was determined on a clinical platform scale (Combics 1, Satorius, NY, USA). Body height was determined with a wall-mounted stadiometer. Blood samples were obtained from an antecubital vein after the subject had rested in a seated position for 15 min. The samples were analyzed by Mid America Clinical Laboratories (Indianapolis, IN, USA) for a comprehensive metabolic panel (serum glucose, electrolytes, blood urea nitrogen, creatinine, albumin, total protein, and liver enzymes), glycated hemoglobin (HbA1c), and a lipid-lipoprotein panel (total cholesterol, high-density lipoprotein cholesterol, and triglycerides). Low-density lipoprotein-cholesterol (LDL-C) was calculated using the following equation [7]:

LDL-C = (total cholesterol − high-density lipoprotein cholesterol − triacylglycerol/5)
(1)

Subjects without type 2 diabetes were further characterized with normal glucose tolerance or prediabetes based on their screening visit HbA1c levels (Normal: HbA1c < 5.7%, prediabetes: 5.7% ≤ HbA1c < 6.5%) according to the American Diabetes Association [8]. Individuals with type 2 diabetes diagnosed more than 6 months prior and with HbA1c levels < 7.5% were recruited to provide pilot data. Subjects treated with medications, but not insulin were included. Upon completion of the screening process, each subject’s self-reported medical history and blood assessment results were reviewed and approved by the study physician to proceed with the study. Prior to beginning the study, subjects provided written informed consent and all study procedures were approved by the Purdue University Biomedical Institutional Review Board. Subjects were provided a monetary stipend for participating in the study. The trial was registered at clinicaltrials.gov as #NCT01951287.

### 2.3. Experimental Design

The protocol took 7–8 weeks for each subject to complete. During week 1 and upon completion of the study, subjects completed three multi-pass 24-h dietary recalls with a registered dietitian to assess habitual dietary intake and body composition was measured using the BOD POD Gold Standard Body Composition Tracking System (COSMED USA Inc., Concord, CA, USA). Starting from week 2, on six mornings, each separated by at least 6 days, subjects consumed a standard breakfast meal with either 240 mL of water (control), reduced-energy orange juice (Trop50^®^, Tropicana Products, Inc., Bradenton, FL, USA), coffee (sweetened with 12 g table sugar), fat-free milk, low-fat milk, or whole milk in a randomized, crossover manner. The authors were blinded to the test beverages provided on the testing day until after all testing and sample analyses were performed. However, the subjects and kitchen staff members were not blinded to the beverage consumed. Blood samples were collected and analyzed for plasma glucose and insulin concentrations before the meal and every 30 min after the completion of the meal for 4 h.

### 2.4. Multi-Pass Dietary Recall

During week 1, each subject’s customary food intake was estimated using 24-h, multi-pass dietary recalls on 3 days (one weekend day and two non-consecutive weekdays) and analyzed by a research dietitian using the Nutrition Data System for Research software (NDSR 2012; Nutrition Coordinating Center, Minneapolis, MN, USA). Three additional dietary recalls were completed during or soon after the week of the final trial to evaluate potential changes in habitual diet during the study period.

### 2.5. Body Mass and Body Composition

Each subject’s fasting body mass (with light clothing) was measured at the screening evaluation and the morning of each testing day using a digital platform scale. Standing height without shoes was measured with a wall-mounted stadiometer. Body mass index (BMI; kg/m^2^) was calculated from body mass and height measurements. Whole body fat and fat-free masses were estimated by air displacement plethysmography (BOD POD Gold Standard Body Composition Tracking System) (COSMED USA Inc., Concord, CA, USA) at the beginning and end of the study to ensure that no changes in body composition occurred that might confound study outcomes.

### 2.6. Diet

Background diet: Subjects were instructed to maintain their habitual food intake during the study except for the day before each testing day, when the 24-h controlled diet was provided. 24-h controlled diet: All subjects were provided the same standardized meals and beverages to completely consume the day prior to each testing day. These meals were designed to meet 100% of the subjects’ estimated daily energy requirement (Sample menu shown in Supplementary Materials Table S1). Subjects were instructed not to consume any foods or energy-containing beverages that were not provided on these days. Standard breakfast meal: the breakfast consisted of a ham and scrambled egg sandwich and toast with jelly (422 kcal, 48 g carbohydrate) (Table 1 and Table S2).

### 2.7. Acute Meal Glucose Tolerance Test (MGTT)

On each testing day, an intravenous catheter was placed in an antecubital vein by a trained phlebotomist. Subjects then rested in a supine position for 15 min and a fasting blood sample was drawn. Subjects then sat up and consumed a standard breakfast meal with 240 mL of one of the following test drinks: water, reduced-energy orange juice, sugar-sweetened coffee, fat-free milk, low-fat milk, or whole milk. Subjects were asked to consume the entire meal in 10 min and to have consumed at least 120 mL of the beverage by 5 min. The test beverages were commercially available products: Trop50^®^ orange juice (pulp-free, not fortified with calcium or vitamin D); commercial ground caffeinated coffee freshly brewed and sweetened with table sugar, and fluid milks. After consuming the breakfast, blood samples were obtained from the catheter every 30 min for 4 h (~6 mL). The intravenous catheter was flushed with approximately 5 mL 0.9% saline solution after each blood draw to prevent clotting. The subjects were asked to remain awake in the seated position for the duration of the test and allowed to stand up when needing to visit the restroom. Subjects were allowed to read and use electronic devices for entertainment. The blood samples were centrifuged at 4400 rpm at 4 °C for 15 min, and plasma was separated and stored at −80 °C until thawed at the time of analysis.

### 2.8. Plasma Glucose and Insulin Analyses and Calculations

Plasma glucose concentrations were measured by enzymatic colorimetry via an oxidase method (COBAS^®^ Integra 400 analyzer, Roche Diagnostic Systems, Indianapolis, IN, USA). Plasma insulin concentrations were measured using an electrochemiluminescence immunoassay method on the Elecsys^®^ 2010 analyzer (Roche Diagnostic Systems, Indianapolis, IN, USA). All plasma samples from each subject were analyzed on the same day to eliminate between-day analytical variability. The homeostatic model assessment-insulin resistance (HOMA-IR) was calculated using: (fasting glucose (mg/dL) × fasting insulin (µU/mL))/405 and pancreatic β cell function (HOMA-β, %) was calculated using [9]:

(360 × fasting insulin (µU/mL))/(fasting glucose (mg/dL) − 65)
(2)

Positive incremental areas under the curve (AUC) for plasma glucose and insulin were calculated using the trapezoidal rule for periods 0–2 h, 2–4 h and 0–4 h [10] with postprandial glucose and insulin concentrations that were baseline-corrected (by subtracting their respective fasting values from values at each time point).

### 2.9. Sample Size Estimation

Due to the novelty of the research design, there were no ideal studies from which to perform our sample size estimation for determining the effects of iso-volumetric, iso-carbohydrate beverages on plasma glucose and insulin responses to a mixed meal. Therefore, sample size estimation for the current research was based on glucose responses to a white bread meal with either 200 mL of water or whole milk among subjects without type 2 diabetes [11]. Despite an increased amount of energy and carbohydrates in the meal consumed with milk, there was a non-significant trend towards a reduced glucose AUC after consuming the meal with milk (68.8 ± 8.1 mmol/L·min, Mean ± SEM) compared to water (86.5 ± 13.3 mmol/L·min) [11]. Using these results, we estimated that a sample size of 40 subjects would provide ≥80% power at α = 0.05 with correlation *r* = 0.7 to detect an effect size of 0.58 for the differential postprandial glucose AUC between test drinks.

### 2.10. Data Analysis

All data were separately entered into spreadsheets by two research staff members (double-entry) and cross-checked and corrected by the first author. Independent Student’s *t*-test was performed to compare baseline characteristics between groups of subjects with normal glucose tolerance and prediabetes. Two-way repeated measures ANOVA was used to assess the main effects of beverage intake, glucose tolerance condition (normal or prediabetes), and their interaction on glucose and insulin AUCs and peak glucose and insulin concentrations using statistical analysis systems (SAS) mixed model (SAS version 9.3, Cary, NC, USA) for each specific aim. The treatment-specific average peak glucose and insulin concentrations were calculated from the timing of each subject’s peak response, not uniformly, at time 40 min. Tukey adjustments were used for *post hoc* pairwise comparisons. We observed no interaction effect of beverage and glucose tolerance condition on glucose and insulin outcomes. As a result, we are presenting the glucose and insulin responses using data from subjects with normal glucose tolerance and prediabetes as a whole (*n* = 46) in order to achieve the expected power. Since the estimated sample size was based on power calculations designed to determine the main effects of beverage, but not to delineate the beverage × glucose tolerance condition interaction effect, we deem this analysis appropriate. Data for the two groups of subjects separately are included in the Supplementary Materials Figures S1–S4 for reference.

Dietary intake and body composition data pre- and post-study were compared using paired Student’s *t*-tests. All data are presented as least-square mean (lsmean) ± standard error of the lsmean (SEM) based on the ANOVA results, unless otherwise noted, and statistical significance was assigned at *p* < 0.05.

## 3. Results

### 3.1. Baseline Subject Characteristics

Among the 46 subjects, average age and BMI were 50 years and 32.5 kg/m^2^, respectively, and fasting blood glucose and HbA1c levels were clinically normal (Table 2). Twenty-nine subjects had normal glucose tolerance and seventeen had prediabetes based on their screening blood HbA1c levels. As expected, HbA1c and fasting blood glucose were higher among individuals with prediabetes (*p* < 0.05).

### 3.2. Dietary Intake and Body Composition before and at the End of the Study

Dietary intakes of total energy, total carbohydrate, total fat, saturated fat, total protein, and total fiber were not different before and at the end of the study (all subjects, or subjects with normal glucose tolerance or prediabetes separately); neither were subjects’ percentage fat mass and fat-free mass different (Table S3).

### 3.3. Fasting State Glucose Homeostasis on Testing Days

Fasting values of glucose, insulin, HOMA-IR, and HOMA-beta were not different among the testing days for each test beverage (Table S4).

### 3.4. Low-Fat Milk vs. Sugar-Sweetened Coffee vs. Reduced-Energy Orange Juice

**Figure 2:**
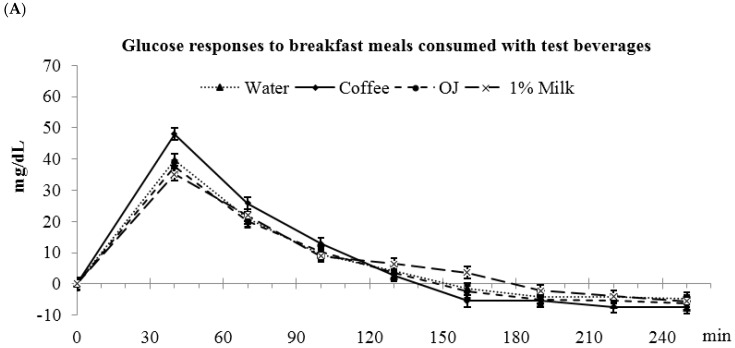

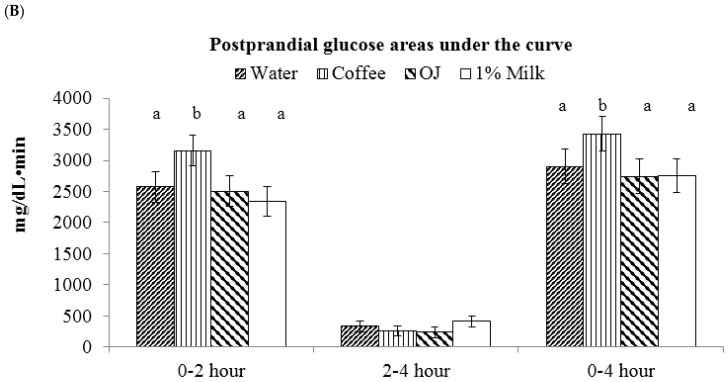
Postprandial plasma glucose responses to breakfast meals consumed with test beverages. (**A**) Changes in glucose concentrations during meal glucose tolerance tests; (**B**) Glucose incremental area under the curve. ^a,b^ Bars with different letters (during the same time frame) were significantly different, *p* < 0.05.

Glucose AUC_(0–4 h)_ was higher for coffee compared to OJ, water, and milk (water: 2903 ± 274 ^a^, coffee: 3425 ± 275 ^b^, OJ: 2741 ± 275 ^a^, milk: 2752 ± 274 ^a^ mg/dL·min, abc: values with different letters were significantly different, *p* < 0.05) (Figure 2). These differences in glucose responses among the beverages were also significant for the glucose AUC_(0–2 h)_, but not the glucose AUC_(2–4 h)_. The peak glucose value was higher for coffee compared to OJ, water and milk (water: 41.8 ± 2.7 ^a^, coffee: 50.3 ± 2.8 ^b^, OJ: 39.7 ± 2.8 ^a^, milk: 36.7 ± 2.7 ^a^ mg/dL, *p* < 0.05).

**Figure 3:**
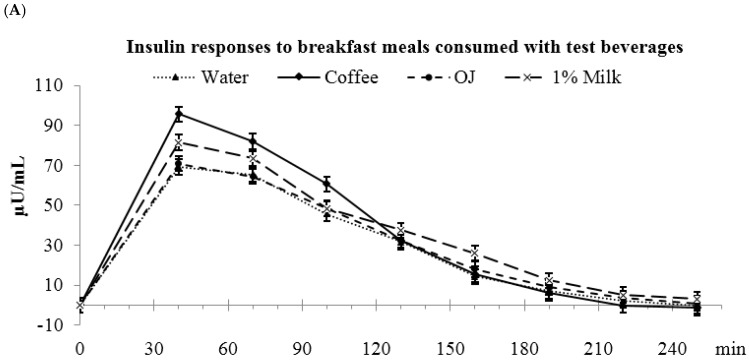

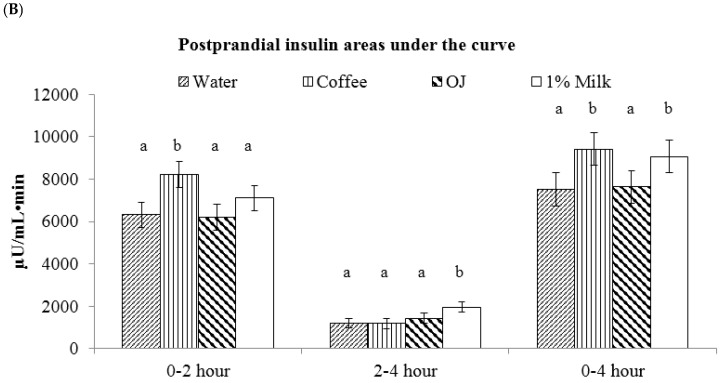
Postprandial plasma insulin responses to breakfast meals consumed with test beverages. (**A**) Changes in insulin concentrations during meal glucose tolerance tests; (**B**) Insulin incremental areas under the curve. ^a,b^ Bars with different letters (during the same time frame) were significantly different, *p* < 0.05.

Insulin AUC_(0–4 h)_ was higher for coffee and milk vs. OJ and water (water: 7515 ± 772 ^a^, coffee: 9406 ± 774 ^b^, OJ: 7635 ± 773 ^a^; milk: 9068 ± 775 ^b^ µU/mL·min, *p* < 0.05) (Figure 3). During the first 2 h after meal consumption, coffee had higher insulin AUC_(0–2 h)_ than OJ , water, and milk (water: 6312 ± 599 ^a^, coffee: 8217 ± 601 ^b^, OJ: 6201 ± 599 ^a^, and milk: 7109 ± 601 ^a^ µU/mL·min, *p* < 0.05). During the 2–4 h period, milk had the highest insulin AUC_(2–4 h)_ (water: 1203 ± 236 ^a^, coffee: 1188 ± 236 ^a^, OJ: 1433 ± 236 ^a^, milk: 1959 ± 236 ^b^ µU/mL·min, *p* < 0.05). The peak insulin value was highest for coffee compared to OJ, water and milk (water: 83.8 ± 7.7 ^a^, coffee: 110.2 ± 7.7 ^b^, OJ: 80.6 ± 7.7 ^a^, milk: 90.1 ± 7.6 ^a^ µU/mL, *p* < 0.05).

### 3.5. Fat-Free vs. Low-Fat vs. Whole Milk

**Figure 4:**
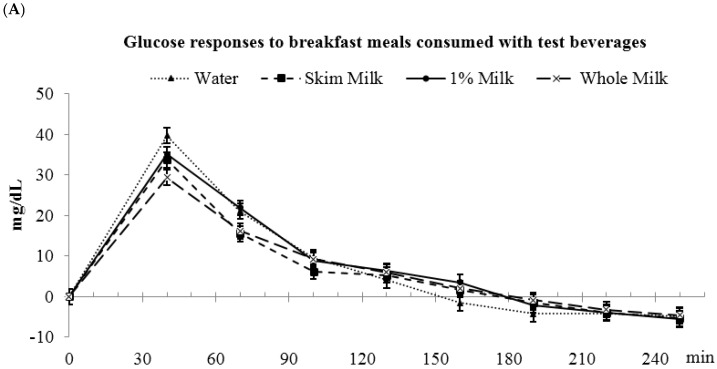

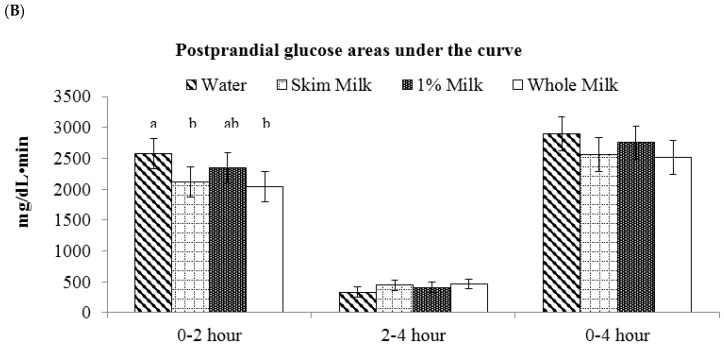
Postprandial plasma glucose responses to breakfast meals consumed with test beverages. (**A**) Changes in glucose concentrations during meal glucose tolerance tests; (**B**) Glucose incremental area under the curve. ^a,b^ Bars with different letters (during the same time frame) were significantly different, *p* < 0.05.

Postprandial glucose AUC_(0–4 h)_ were not different among the beverages (water: 2903 ± 274, fat-free milk: 2559 ± 274, low-fat milk: 2752 ± 274, whole milk: 2509 ± 274 mg/dL·min) (Figure 4). During the first 2 h, glucose AUC_(0–2 h)_ was higher for water vs. fat-free milk and whole milk (water: 2571 ± 244 ^a^, fat-free milk: 2115 ± 244 ^b^, low-fat milk: 2342 ± 244 ^a,b^, and whole milk: 2043 ± 244 ^b^ mg/dL·min, *p* < 0.05). No differences in glucose AUC_(2–4 h)_ were observed during the 2–4 h period. The peak glucose concentrations were not different among fat-free milk, low-fat, and whole milk, but whole milk was lower than the water control (water: 41.9 ± 2.7 ^a^, fat-free milk: 37.4 ± 2.8 ^a,b^, low-fat milk: 36.7 ± 2.7 ^a,b^, whole milk: 31.5 ± 2.7 ^b^, *p* < 0.05).

**Figure 5:**
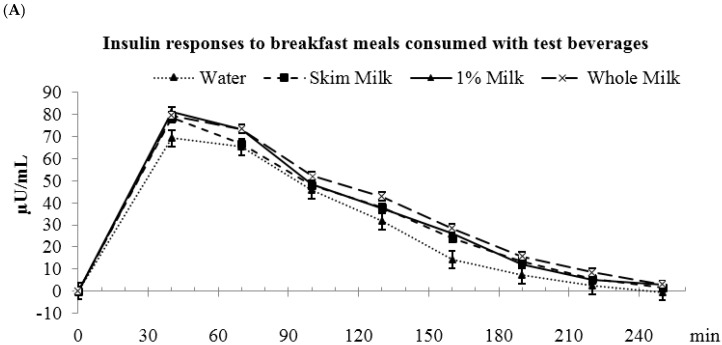

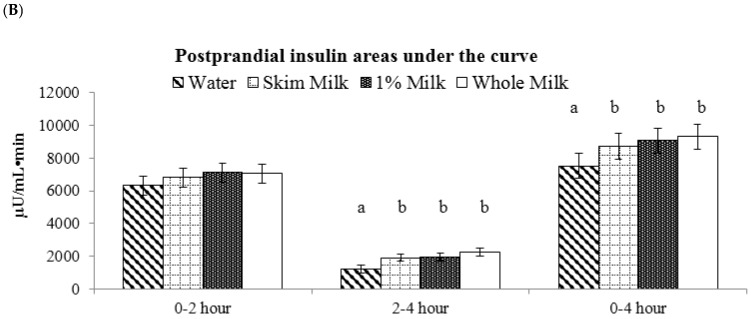
Postprandial plasma insulin responses to breakfast meals consumed with test beverages. (**A**) Changes in insulin concentrations during meal glucose tolerance tests; (**B**) Insulin incremental area under the curve. ^a,b^ Bars with different letters (during the same time frame) were significantly different, *p* < 0.05.

Insulin AUC_(0–4 h)_ was lower for water compared to the milk beverages (water: 7515 ± 772 ^a^, fat-free milk: 8719 ± 772 ^b^, low-fat milk: 9068 ± 774 ^b^, and whole milk: 9315 ± 772 ^b^ µU/mL·min, *p* < 0.05) (Figure 5). During the first 2 h, insulin AUCs were not different among water and milks. During the 2–4 h period, water had lower insulin AUC_(2–4 h)_ than the milk beverages (water: 1203 ± 236 ^a^, fat-free milk: 1909 ± 236 ^b^, low-fat milk: 1959 ± 236 ^b^, whole milk: 2262 ± 236 ^b^ µU/mL·min, *p* < 0.05). The peak insulin concentrations were not different among water and the milks (water: 83.8 ± 7.7, fat-free milk: 90.8 ± 7.6, low-fat milk: 91.9 ± 7.7, and whole milk: 89.9 ± 7.7 µU/mL).

## 4. Discussion

The current study compared the glucose and insulin responses to common breakfast beverages with equal carbohydrate quantity consumed with a standard breakfast meal. We hypothesized that due to the differences in glycemic indices among sugar-sweetened coffee, reduced-energy orange juice, and low-fat milk, the postprandial glucose responses to the test meals from the highest to the lowest would be coffee, OJ, and milk. Consistent with this hypothesis, we observed greater glucose responses to coffee than OJ and milk, but no differences between milk and OJ. With respect to insulin, we observed greater insulin responses to coffee and milk than OJ. These results provide important applicable evidence for the general populous to consider regarding their daily breakfast beverage choices. Our data suggest that for middle-aged adults who are overweight and obese, choosing reduced-energy orange juice or fluid milks are preferable options than sugar-sweetened coffee with regards to postprandial glucose responses.

### 4.1. Aim 1: Low-Fat Milk vs. Sugar-Sweetened Coffee vs. Reduced-Energy Orange Juice

One of the major determinants of postprandial glucose response is the type of carbohydrate consumed. In this study, the beverages differ in their carbohydrate source: the carbohydrate source of coffee was sucrose (1:1 molar ratio between glucose and fructose), of reduced-energy orange juice was a mixture of fructose/sucrose/glucose/starch, and of milk was lactose (1:1 molar ratio between glucose and galactose). Using white bread as a reference, the GIs of these carbohydrates by themselves in coffee, OJ, and milk are approximately 103, 88 (average of glucose/sucrose/fructose), and 67, respectively [5]. However, our results showed that the postprandial glucose responses to the beverages containing these carbohydrates only partly comply with the order of these glycemic indices.

In addition to the carbohydrate, other components in the test beverages may also impact postprandial glucose control. The higher glucose responses to sugar-sweetened coffee may also be partially explained by the caffeine content. Several previous studies consistently showed that caffeine consumption induced an acute reduction in insulin sensitivity among individuals who were healthy, obese, or had type 2 diabetes [12,13,14]. These studies showed that consuming caffeinated coffee with a meal or a glucose solution promoted higher glucose responses (16% to 47%) compared to the control drink. Thus, the elevated postprandial glucose response to coffee may be explained by both its high glycemic index carbohydrate source (sucrose) and caffeine content.

Similarly, the non-carbohydrate component of milk, specifically the proteins present in milk, may contribute to the relatively modest glucose response to milk. Previous studies showed that fluid milk has relatively low glycemic index (~30–40) but high insulin responses (~90–148) [6,15]. This phenomenon was described as a “dissociation of the glycemic and insulinemic responses” [6] and may be explained by both its lactose and protein components [16]. Lactose has a moderately low GI of approximately 67, and the whey and casein proteins in milk may further function to reduce the glucose response by reducing the gastric emptying rate as well as stimulating insulin secretion for glucose clearance, resulting in a further reduced GI of ~30-40 for milk [16,17]. Our results are consistent with this dissociation phenomenon. We observed that drinking milk with a meal did not further increase the glucose response but increased the insulin responses compared to orange juice and water. It may be speculated that the ingested milk slowed the gastric emptying of the entire meal as well as induced insulin secretion to promote the clearance of the additional glucose from circulation. 

We observed no difference in glucose responses between orange juice and the water control co-consumed with a complex meal, despite of the additional 12 g of carbohydrate. Limited studies exist on the postprandial glucose responses to orange juice consumed with a meal [18]. Ghanim et al. reported lower blood glucose concentrations at 1 h after consuming regular orange juice (300 kcal, 75 g carbohydrates) with a high-fat, high carbohydrate meal (900 kcal, 81 g carbohydrates, 51 g fat, and 32 g protein) than water control among healthy, normal-weight adults [18]. One limitation of Ghanim’s study is that they measured blood glucose concentrations starting from 1 h after meal ingestion when blood glucose had already returned to fasting concentrations, thus missing the trend of glucose response during the first hour. Despite the many differences in study design between the current study and that of Ghanim et al., such as subjects included and test meal/beverage composition, these results support that consuming orange juice with a meal does not further increase glucose responses. Little is known regarding the underlying mechanism. While the anti-oxidant/inflammatory properties [18] and pectin content [19] of orange juice may be speculated to impact glucose responses, postprandial glucose control is a multifaceted process, and future studies are warranted to investigate potential mechanisms and the effect of consuming OJ compared to beverages with comparable carbohydrate quantities.

It needs to be addressed that compared to water, consuming OJ and low-fat milk in addition to the breakfast sandwich did not further increase glucose responses despite the additional carbohydrate content. These observations may be explained by some known and unknown properties of OJ and milk themselves as discussed above. Also, it is probable that consuming these beverages with a mixed-meal may have masked the differences in the glucose responses to milk, OJ, and water if they were consumed alone. Lastly, with respect to energy intake as well as postprandial glucose control, water may be the most preferable beverage. In our study consuming water at the breakfast meal lead to equally low glucose responses as milk and OJ without the added energy intake.

### 4.2. Aim 2: Fat-Free vs. Low-Fat vs. Whole Milk

We observed no differences in glucose and insulin responses after the meal was consumed with milk containing different amounts of fat. Our results extend previous research documenting comparable glucose and insulin responses after fat-free milk and whole milk were consumed alone [6].

The current study has strengths and limitations. For strength, we specifically recruited middle-aged subjects who were overweight and obese and have a higher risk for developing type 2 diabetes. The results can be readily applied to this population. Several strategies were utilized to ensure consistency in subjects’ metabolic states and dietary habits during the study: (1) we provided standard meals for subjects to consume before each testing day; (2) we measured subjects’ pre and post body compositions and dietary intakes and detected no changes for the subjects. In addition, the authors were blinded to the test beverages until sample analyses were finished to avoid potential bias. We acknowledge that the study also has several limitations. We recruited subjects without regard to their habitual beverage intake, the effect of which on the acute postprandial glucose control is unknown. Another limitation is the lack of measurements of parameters such as incretin hormones, C-peptide, and rate of gastric emptying, which may provide insight for the underlying mechanisms. We measured glucose and insulin concentrations every 30 min during the 4-h postprandial period. A more frequent blood sampling, i.e., every 15 min, during the first hour would have provided a more detailed profile of early phase glucose and insulin responses. Previous studies suggested potential second meal effects of different beverages consumed [4,18], thus a longer testing period with a second meal would reveal whether such effects exist for the test beverages. Lastly, we did not include a treatment arm where coffee was consumed with condiments other than the table sugar, such as dairy creamers (e.g., fluid milks, half and half) and non-dairy creamers (e.g., soy milk, artificial creamers). Testing additional treatments may be of interest for future research, but would have greatly increased the burden on these participants who had already completed six trials.

## 5. Conclusions

Common breakfast beverages containing the same amount of digestible carbohydrate elicit different postprandial glycemic and insulinemic responses when consumed with a meal. Low-fat milk and reduced-energy orange juice may be preferred beverages to consume with a meal compared to sugar-sweetened coffee for people who are at risk of type 2 diabetes. Additionally, milks varying in fat content are comparable options with regards to postprandial glycemic responses.

## Figures and Tables

**Table 1:** Nutrient composition of test drinks and meals

Beverage Consumed with Meal	Beverage Only						Whole Meal (Beverage + Sandwich)		
	Energy (kcal)	Total CHO * (g)	CHO Source	Dietary Fiber (g)	Fat (g)	Protein (g)	Energy (kcal)	Total CHO (g)	Dietary Fiber
Water (Control)	0	0	NA	0	0	0	422	48	2.0
Sugar-Sweetened Coffee	50	12	Sucrose	0.1	0.1	0.3	472 (422 + 50)	60	2.1
Orange Juice (Trop 50)	50	12	Sucrose, fructose, glucose	0.2	0	0.6	472 (422 + 50)	60	2.2
Fat-Free Milk	82	12	Lactose	0	0	8	504 (422 + 82)	60	2.0
Low-Fat Milk	101	12	Lactose	0	2	8	523 (422 + 101)	60	2.0
Whole Milk	159	12	Lactose	0	9	9	581 (422 + 159)	60	2.0

* CHO: carbohydrate.

**Table 2:** Subject characteristics.

Parameter	All * (*n* = 46)	Normal (*n* = 29)	Prediabetes (*n* = 17)	*p* ^#^
Age (year)	50 ± 1.4	50 ± 1.8	49 ± 2.5	0.85
Gender	33F/13M	20F/9M	13F/4M	
BMI ^&^ (kg/m^2^)	32.5 ± 0.7	32.2 ± 0.8	33.0 ± 1.2	0.57
Glucose, serum (mg/dL)	94.0 ± 1.4	89.6 ± 1.5	95.3 ± 2.0	0.02
Glycated hemoglobin A1c (%) ^‡^	5.6 ± 0.04	5.4 ± 0	5.9 ± 0.1	<0.0001

* Data are Mean ± SEM; ^#^ Independent *t*-test between normal and prediabetes; ^&^ BMI: body mass index; ^‡^ Normal: HbA1c < 5.7%, prediabetes: 5.7% ≤ HbA1c < 6.5%.

## Supplementary Materials

The following are available online at http://www.mdpi.com/2072-6643/9/1/32/s1, Figure S1: Postprandial plasma glucose responses to breakfast meals consumed with test beverages among subjects with normal glucose tolerance. ^a,b^: Bars with different letters (during the same time frame) were significantly different, *p* < 0.05, Figure S2: Postprandial plasma insulin responses to breakfast meals consumed with test beverages among subjects with normal glucose tolerance. ^a,b^: Bars with different letters (during the same time frame) were significantly different, *p* < 0.05, Figure S3: Postprandial plasma glucose responses to breakfast meals consumed with test beverages among subjects with normal glucose tolerance. ^a,b^: Bars with different letters (during the same time frame) were significantly different, *p* < 0.05, Figure S4: Postprandial plasma insulin responses to breakfast meals consumed with test beverages among subjects with normal glucose tolerance. ^a,b^: Bars with different letters (during the same time frame) were significantly different, *p* < 0.05, Table S1: Sample meals and macronutrients distributions for the 24-h controlled diet before each test day based on a 2000-calorie diet, Table S2: Nutrient composition of test breakfast sandwich, Table S3: Subjects’ dietary intakes and body compositions before and at the end of the study, Table S4: Fasting state glucose homeostasis on the test mornings for each beverage.

## Abbreviations

The following abbreviations are used in this manuscript:

AUC	Incremental area under the curve
OJ	Reduced-energy orange juice
HOMA-IR	Homeostatic model assessment-Insulin resistance
HOMA-β	Homeostatic model assessment-β cell function
