# Supplementary material for: Postprandial Glycemic and Insulinemic Responses to Common Breakfast Beverages Consumed with a Standard Meal in Adults Who Are Overweight and Obese
Source: Nutrients. 2017 Jan 4;9(1):32. doi: 10.3390/nu9010032 (PMC5295076; doi:10.3390/nu9010032)
Supplement: Supplementary file 1 [file nutrients-09-00032-s001.docx]

Supplementary Materials: Postprandial Glycemic and Insulinemic Responses to Common Breakfast Beverages Consumed with a Standard Meal in Adults Who Are Overweight and Obese

Jia Li, Elsa Janle and Wayne W. Campbell


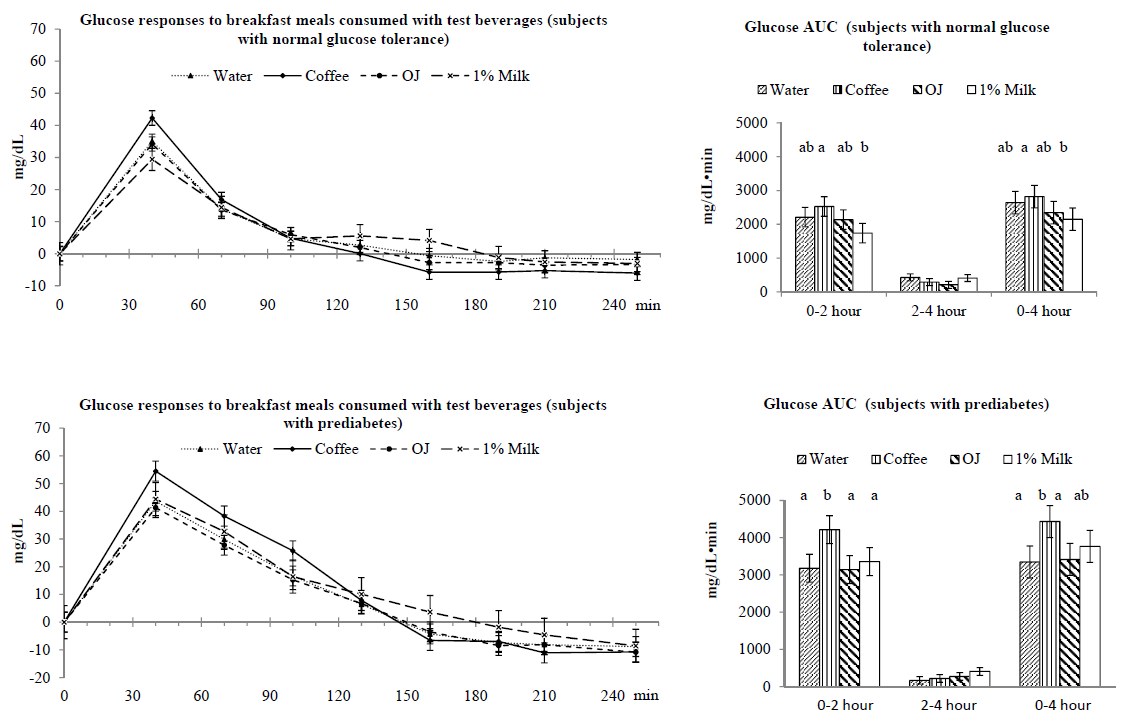


**Figure S1.** Postprandial plasma glucose responses to breakfast meals consumed with test beverages among subjects with normal glucose tolerance. Bars with different letters (during the same time frame) were significantly different, *p* < 0.05.


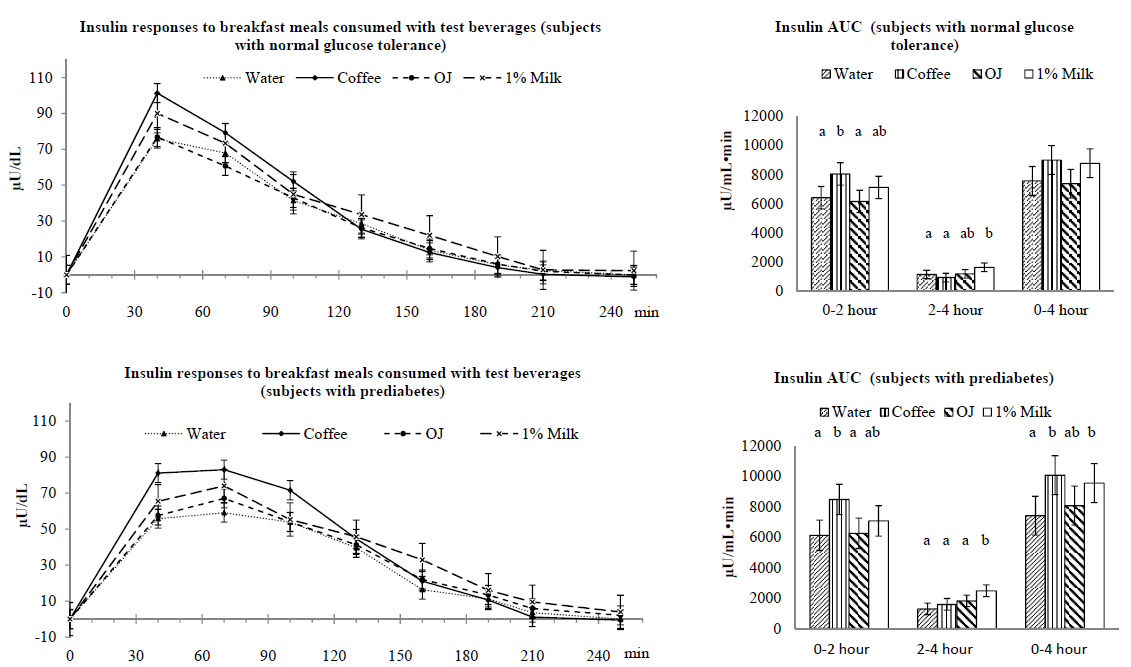


**Figure S2.** Postprandial plasma insulin responses to breakfast meals consumed with test beverages among subjects with normal glucose tolerance. Bars with different letters (during the same time frame) were significantly different, *p* < 0.05.


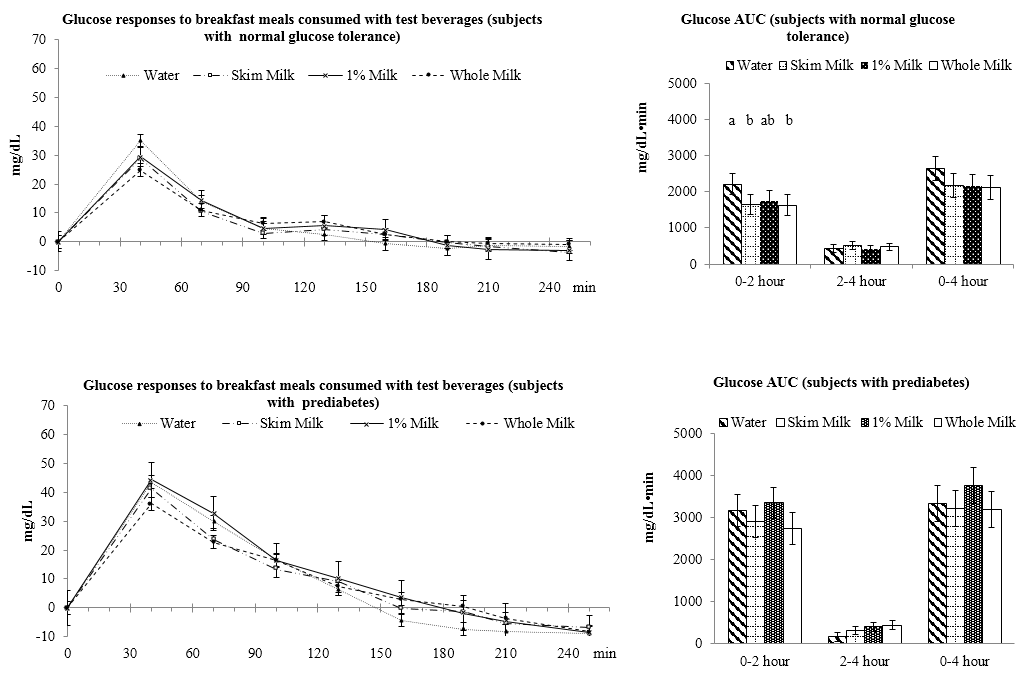


**Figure S3.** Postprandial plasma glucose responses to breakfast meals consumed with test beverages among subjects with normal glucose tolerance. Bars with different letters (during the same time frame) were significantly different, *p* < 0.05.


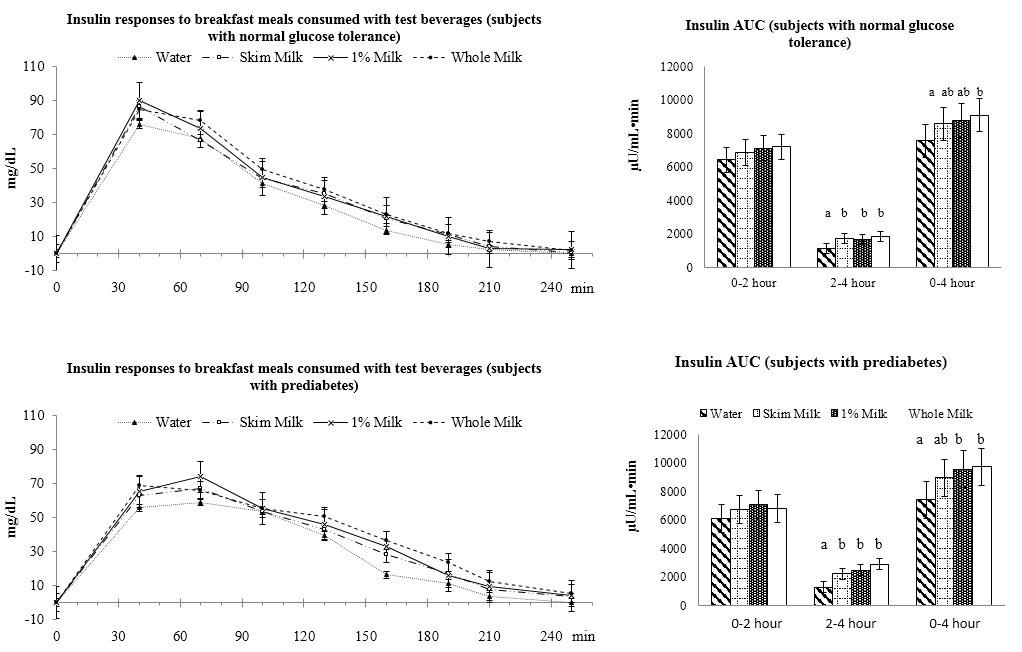


**Figure S4.** Postprandial plasma insulin responses to breakfast meals consumed with test beverages among subjects with normal glucose tolerance. Bars with different letters (during the same time frame) were significantly different, *p* < 0.05.

**Table S1.** Sample meals and macronutrients distributions for the 24-h controlled diet before each test day based on a 2000-calorie diet.

| Meal/Menu | Total Calories, Kcal | Carbohydrate, g (% E) | Fat, g (% E) | Protein, g (% E) |
| --- | --- | --- | --- | --- |
| **Breakfast** | 532 | 65 g (49%) | 24 g (41%) | 13 g (10%) |
| Blueberries |  |  |  |  |
| Buttermilk Pancakes |  |  |  |  |
| Butter, salted |  |  |  |  |
| Breakfast Syrup |  |  |  |  |
| Apple Juice |  |  |  |  |
| Turkey Bacon |  |  |  |  |
| **Lunch** | 581 | 68 g (47%) | 23 g (36%) | 24 g (17%) |
| Diet Soda |  |  |  |  |
| Chicken Breast Patties |  |  |  |  |
| Swiss Cheese Slices |  |  |  |  |
| Salad dressing, |  |  |  |  |
| Sandwich buns |  |  |  |  |
| Vegetables with Marinara Sauce |  |  |  |  |
| mandarin oranges |  |  |  |  |
| **Afternoon Snacks** | 299 | 44 g (60%) | 10 g (30%) | 11 g (10%) |
| Popcorn |  |  |  |  |
| Diet soda |  |  |  |  |
| 1% milk |  |  |  |  |
| Granola bar |  |  |  |  |
| **Dinner** | 577 | 64 g (44%) | 16 g (25%) | 44 g (31%) |
| Whole Grain Instant Brown Rice |  |  |  |  |
| Beef, tenderloin |  |  |  |  |
| Snowpeas |  |  |  |  |
| Soy sauce |  |  |  |  |
| Mixed vegetables |  |  |  |  |
| Fat Free Devil’s Food Cookie Cakes |  |  |  |  |
| Broccoli in Cheese |  |  |  |  |
| Total | 1990 | ~241 g (48%) | ~73 g (34%) | 92 g (18%) |

**Table S2.** Nutrient composition of test breakfast sandwich.

| Item | Energy (kcal) | Total CHO (g) | Dietary Fiber (g) | Fat (g) | Protein (g) |
| --- | --- | --- | --- | --- | --- |
| Cooked ham (44 g) | 99 | 0 | 0 | 7 | 9 |
| Scrambled whole egg (39 g) | 54 | 1 | 0 | 4 | 4 |
| White bread (75 g) | 199 | 37 | 2 | 2 | 6 |
| Margarine (5 g) | 30 | 0 | 0 | 3 | 0 |
| Jelly (14 g) | 40 | 10 | 0 | 0 | 1 |
| Total | 422 | 48 | 2 | 16 | 20 |

**Table S3.** Subjects’ dietary intakes and body compositions before and at the end of the study.

|  | Group | Before ^&^ | End ^&^ | *p* Value * |
| --- | --- | --- | --- | --- |
| **Dietary intake** |  |  |  |  |
| Total energy intake (kcal/day) | All | 1899 ± 69 | 1805 ± 74 | 0.19 |
|  | Normal ^$^ | 1905 ± 90 | 1805 ± 97 | 0.28 |
|  | Prediabetes ^$^ | 1890 ± 111 | 1802 ± 113 | 0.50 |
| Carbohydrate intake (g/day) | All | 228 ± 9 | 214 ± 11 | 0.16 |
|  | Normal | 231 ± 13 | 213 ± 13 | 0.19 |
|  | Prediabetes | 224 ± 14 | 215 ± 20 | 0.62 |
| Carbohydrate intake (%) | All | 46 ± 1 | 46 ± 1 | 0.51 |
|  | Normal | 47 ± 1 | 46 ± 1 | 0.42 |
|  | Prediabetes | 47 ± 1 | 45 ± 1 | 0.92 |
| Protein intake (g/day) | All | 76 ± 3 | 74 ± 3 | 0.38 |
|  | Normal | 75 ± 4 | 76 ± 5 | 0.78 |
|  | Prediabetes | 78 ± 6 | 70 ± 3 | 0.15 |
| Protein intake (%) | All | 16 ± 1 | 17 ± 1 | 0.14 |
|  | Normal | 16 ± 1 | 17 ± 1 | 0.14 |
|  | Prediabetes | 16 ± 1 | 16 ± 1 | 0.64 |
| Total fat intake (g/day) | All | 76 ± 3 | 72 ± 3 | 0.32 |
|  | Normal | 74 ± 4 | 71 ± 4 | 0.48 |
|  | Prediabetes | 78 ± 5 | 74 ± 5 | 0.50 |
| Fat intake (%) | All | 35 ± 1 | 35 ± 1 | 0.89 |
|  | Normal | 34 ± 1 | 34 ± 1 | 0.76 |
|  | Prediabetes | 34 ± 1 | 36 ± 1 | 0.81 |
| Saturate fat intake (g/day) | All | 26 ± 1 | 25 ± 1 | 0.34 |
|  | Normal | 26 ± 2 | 24 ± 2 | 0.26 |
|  | Prediabetes | 26 ± 3 | 26 ± 2 | 0.97 |
| Total dietary fiber (g/day) | All | 17 ± 1 | 16 ± 1 | 0.24 |
|  | Normal | 17 ± 1 | 16 ± 1 | 0.31 |
|  | Prediabetes | 17 ± 1 | 16 ± 1 | 0.58 |
| **Body composition** |  |  |  |  |
| Fat mass, % | All | 42.8 ± 1.2 | 43.0 ± 1.2 | 0.75 |
|  | Normal | 42.9 ± 7.4 | 43.7 ± 7.0 | 0.76 |
|  | Prediabetes | 42.6 ± 8.9 | 42.7 ± 8.6 | 0.90 |
| Fat-free mass, % | All | 57.1 ± 1.2 | 57.0 ± 1.2 | 0.75 |
|  | Normal | 57.1 ± 7.4 | 57.0 ± 7.0 | 0.76 |
|  | Prediabetes | 57.4 ± 9.0 | 57.4 ± 8.6 | 0.90 |

^&^ Data are Mean ± SEM; * Paired *t*-test before and at the end of the study; ^$^ Subjects were categorized into two groups: Normal: HbA1c <5.7%, prediabetes: 5.7% ≤ HbA1c < 6.5%).

**Table S4.** Fasting state glucose homeostasis on the test mornings for each beverage.

|  | Water | Coffee | OJ | Fat-Free Milk | Low-Fat Milk | Whole Milk |
| --- | --- | --- | --- | --- | --- | --- |
| Fasting glucose (mg/dL) * | 91.7 ± 1.5 | 93.6 ± 1.5 | 92.3 ± 1.5 | 92.3 ± 1.5 | 92.7 ± 1.5 | 92.3 ± 1.5 |
| Fasting insulin (µU/mL) * | 10.9 ± 0.9 | 12.0 ± 0.9 | 10.5 ± 0.9 | 11.3 ± 0.9 | 10.8 ± 0.9 | 11.6 ± 0.9 |
| HOMA-IR * | 2.5 ± 0.2 | 2.8 ± 0.2 | 2.5 ± 0.2 | 2.5 ± 0.2 | 2.5 ± 0.2 | 2.7 ± 0.2 |
| HOMA-β * | 1.5 ± 0.2 | 1.6 ± 0.2 | 1.4 ± 0.2 | 1.5 ± 0.2 | 1.4 ± 0.2 | 1.5 ± 0.2 |

* Results were from repeated measures ANOVA. There were no differences in each parameter among the testing days.
